# Editorial: Immunobiotics—Interactions of Beneficial Microbes with the Immune System

**DOI:** 10.3389/fimmu.2017.01580

**Published:** 2017-11-17

**Authors:** Julio Villena, Haruki Kitazawa

**Affiliations:** ^1^Immunobiotics Research Group, Tucuman, Argentina; ^2^Laboratory of Immunobiotechnology, Reference Centre for Lactobacilli (CERELA-CONICET), Tucuman, Argentina; ^3^Food and Feed Immunology Group, Laboratory of Animal Products Chemistry, Graduate School of Agricultural Science, Tohoku University, Sendai, Japan; ^4^Livestock Immunology Unit, International Education and Research Center for Food and Agricultural Immunology (CFAI), Graduate School of Agricultural Science, Tohoku University, Sendai, Japan

**Keywords:** immunobiotics, lactic acid bacteria, mucosal immunology, innate immunity, beneficial microbes

**Editorial on the Research Topic**

**Immunobiotics—Interactions of Beneficial Microbes with the Immune System**

The term “immunobiotic” has been proposed to define microbial strains that are able to beneficially regulate the immune system of the host. Over the past few years, we have witnessed the emergence of robust development in the application of immunobiotics to combat infections, and researchers have found that the use of beneficial microbes is an interesting alternative to prevent and reduce the severity of infections in humans and animals. In a study by Villena et al., the advances in the application of immunobiotics for preventing intestinal viral infections are analyzed. The capacity of immunobiotics to beneficially modulate the intestinal activation of toll-like receptor 3 (TLR3) and to reduce the local inflammatory-tissue damage is highlighted. Complementing this article, Albarracín et al. reported that immunobiotics substantially modify the immunotranscriptomic response of intestinal epithelial cells after activation of TLR3, inducing an improvement of type-I interferons and antiviral factors and a differential modulation of cytokines, chemokines, and adhesion molecules. Moreover, Kandasamy et al. reviewed the specific effects of Gram-positive and Gram-negative immunobiotics in modulating intestinal immunity against rotavirus and emphasized that immunomodulatory functions of beneficial microbes are species and strain specific.

The effect of immunobiotics on the gut innate and adaptive immune responses to enteric pathogens has been recognized conclusively. However, the influence of immunobiotics on the immune responses in distal mucosal sites and its impact in the outcome of respiratory infections has recently been exposed. In this regard, some studies have demonstrated the potential of beneficial microbes in enhancing respiratory antiviral immunity. Zelaya et al. provide an update on the modulation of respiratory immunity by immunobiotics, and their impact on influenza virus infection. Interestingly, the article highlights the recent findings demonstrating the capacity of some immunobiotic strains to reduce the severity of viral disease through the regulation of the immune-coagulative responses in the respiratory tract. Research indicates that beneficial microbes would be able to influence not only the outcome of viral infections but also secondary bacterial pneumonia. In this regard, Clua et al. demonstrate that the nasal priming with inactivated immunobiotics or purified peptidoglycan improved the resistance to primary respiratory syncytial virus infection, and secondary pneumococcal pneumonia in infant mice. Researchers show that a differential modulation of lung immune cell populations and cytokine production are involved in the protective effects induced by inactivated immunobiotics. Interestingly, the approach of using immunobiotics for modulating respiratory immune responses can be extended for the protection of immunocompromised hosts, as elegantly reviewed by Salva and Alvarez.

Several research works have also reported that immunobiotic intervention had beneficial effects on chronic inflammatory conditions of the gastrointestinal tract. As reviewed by Carvalho et al. and Shigemori and Shimosato, studies in several animal models have provided evidence of the health benefits of certain bacterial species in the alleviation of intestinal inflammation. It was reported that the beneficial effects induced by immunobiotics could be achieved by several mechanisms including the modulation mucosal cytokine profiles, IgA levels, expression patterns of cell surface molecules of antigen presenting cells, or gut microbiota composition, as shown by Carasi et al. and Bene et al. Strikingly, lactate that has long been considered as a metabolic by-product of cells is now seen as a potential beneficial microbiota metabolite with immunomodulatory functions. In this regard, Iraporda et al. revealed that the local treatment with lactate prevents intestinal inflammation in the TNBS-induced colitis model.

In the past few years, researchers have been trying to genetically improve the beneficial microbes designed to express anti-inflammatory factors such as cytokines and enzymes, and they have used this genetically modified immunobiotics as a promising strategy in the treatment of inflammatory bowel diseases and mucositis (Carvalho et al.; Shigemori and Shimosato). Of note, the use of microbes to alleviate intestinal inflammation has not been limited to classical immunobiotics strains such as lactic acid bacteria. Researchers have started to search new beneficial strains in other sources as shown by two articles in this research topic. Indrelid et al. reported that *Methylococcus capsulatus* prevents experimentally induced colitis in a murine model of inflammatory bowel disease by influencing dendritic cell maturation, cytokine production, and subsequent T-cell activation, proliferation, and differentiation. In addition, Diling et al. demonstrated that a protein isolated from the fungus *Hericium erinaceus* exhibited immunomodulatory activity in LPS-activated macrophages *in vitro* by decreasing the overproduction of inflammatory cytokines. Moreover, *in vivo* studies showed that the immunomodulatory fungal protein reduced intestinal inflammation in TNBS-treated animals.

Intestinal dysbiosis, metabolic endotoxemia, and systemic inflammation have been associated with metabolic disorders, such as obesity, insulin resistance, and type-2 diabetes. In this regard, Leite et al. by performing a clinical trial in type-2 diabetes patients and healthy controls observed that the prevalence of Gram-negative species in the gut and the increased plasma IL-6 could be linked to insulin resistance. On the other hand, alterations of microbiota in other mucosal tissues in type-2 diabetes patients have been less explored. Interestingly, Ling et al. reported for the first time that dysbiosis of the urinary microbiota was associated with increased levels of urinary IL-8 in female type-2 diabetes patients. These and other studies suggest that modulation of microbiota could have the potential to reduce inflammation and diminish the severity of alterations in metabolic disorders. In agreement, Fabersani et al. demonstrated that some immunobiotic Gram-positive strains are able to differentially modulate the production of adipokines and leptin by macrophages and adipocytes. Of note, although most studies of the microbiota influence on metabolic alterations have focused on obesity and diabetes, recent findings show that intestinal dysbiosis could be also implicated in inflammatory and metabolic alterations of other diseases. In the case of systemic lupus erythematosus, Rodríguez-Carrio et al. show that intestinal dysbiosis is associated with altered serum levels of free fatty acids and endothelial activation in these patients, opening the door to a new potential application of immunobiotics that must be explored in-depth.

Research in immunobiotics continues to evolve as many laboratories are employing cutting-edge technology to investigate the complex interactions of these beneficial microorganisms with the immune system (Albarracín et al.; Kang et al.; Adachi et al.). During the past decade, our understanding of immunobiotics–host interaction was profoundly transformed by the discovery of microbial molecules and host receptors involved in the modulation of gut-associated immune system, as well as the systemic and distant mucosal immune systems. The compilation of research articles included in this research topic gives an overview of the recent advances in immunobiotics and help reader to locate them (Ko et al.; Wan et al.; Dong et al.; van Beek et al.; Górska et al.; Yu et al.).

## Author Contributions

All authors have made a substantial, direct, and intellectual contribution to the work and approved the final version of the manuscript for publication.

## Conflict of Interest Statement

The authors declare that the research was conducted in the absence of any commercial or financial relationships that could be construed as a potential conflict of interest.

